# Using online videos to target defeatist beliefs: Changing I can’t to I can with a veteran living with serious mental illness

**DOI:** 10.1016/j.psycr.2023.100169

**Published:** 2023-12

**Authors:** Jay A. Gorman, Brian J. Stevenson, Erin D. Reilly

**Affiliations:** a Clinical Research Psychologist, VISN 1 Mental Illness Research, Education, and Clinical Center (MIRECC), VA Bedford Healthcare System, Bedford, MA, USA; b Assistant Professor of Psychiatry, Boston University School of Medicine, Boston, MA, USA; c Assistant Professor of Psychiatry, UMASS Chan Medical School, Worcester, MA, USA

**Keywords:** Technology, Serious mental illness, Supported employment, Psychosocial rehabilitation, Schizophrenia, Work readiness

## Abstract

Preparing persons diagnosed with Serious Mental Illness (SMI) for vocational rehabilitation can require the coordination of multiple resources and interventions. This case report depicts a supplementary intervention utilizing YouTube to help intervene in the cycle of defeatist beliefs fueling negative symptoms, decrease employment-related anxiety, increase openness to a variety of work responsibilities, and increase job-seeking behaviors. Options for integrating technology into work readiness preparation are also discussed. Quantitative assessment, qualitative information, and a Subjective Units of Distress Scale (SUDS) are used to capture this person’s vocational rehabilitation journey.

## Introduction

Persons living with serious mental illness (SMI) makeup approximately 5.6% of the United States (U.S.) adult population (Substance Abuse and Mental Health Services Administration 2021) and most report employment as a goal (Frounfelker et al., 2010; Ramsay et al., 2011). However, for persons living with SMI, the unemployment rate is around 85% (Rosenheck et al., 2006; Salkever et al., 2007), disproportionately higher and longer lasting compared to the general population (Luciano and Meara, 2014). Comparatively, as of September 2022, the overall unemployment rate in the U.S was only 3.7% (U.S. Department of Labor; Bureau of Labor Statistics 2022). These differences highlight employment discrepancies in the U.S. and speak to the importance of pursuing interventions to increase employment attainment, maintenance, and progression for persons with SMI. The Individual Placement and Support (IPS) model of Supported Employment (SE) is an evidence-based practice (EBP) that has proven efficacious for helping individuals with SMI obtain competitive employment (Bond et al., 2008). However, IPS-SE was designed to support individuals who have clearly identified competitive employment goals, specifically (Roberts and Pratt, 2007). Thus, individuals who struggle with identifying vocational goals, interest, or have unclear motivation related to unhelpful beliefs about one’s employment potential may need additional intervention before they can reap the benefits of vocational rehabilitation services designed to improve competitive employment outcomes.

From a stages of change perspective, individuals without clear vocational interest or work motivation have been described as “pre-contemplative” (Iwanaga et al., 2020) are presumed to need additional intervention before engaging in an EBP (e.g., IPS-SE) to address their vocational needs (Sally Rogers et al., 2004). For example, cognitive variables such as hopelessness, low self-efficacy, and defeatist beliefs negatively impact work functioning (Davis et al., 2004; Grant and Beck, 2009; Hoffmann et al., 2000; Kiwanuka et al., 2014) and likely contribute to non-engagement in employment services; thus, interventions targeting these cognitive variables may support engagement in work-related activities.

One clinical trial specifically sampled a pre-contemplative group of individuals living with SMI who were unemployed and interested in work, but not interested in enrolling in employment services (Russinova et al., 2018). Participants in this study were randomly enrolled to a waitlist control condition or to a peer-led, group-based intervention targeting work hope, self-efficacy, vocational identity, work motivation, and self-stigma. Outcomes demonstrated that 1) negative beliefs are malleable among pre-contemplative individuals living with SMI and 2) improving negative cognitions leads to enhanced vocational outcomes and increases engagement in employment services (Russinova et al., 2018). Further, for individuals living with SMI who engage in cognitive-behavioral therapy and more specifically, cognitive restructuring techniques, report an improvement in both symptoms and functioning [(Turkington et al., 2006; Mueser et al., 2015) respectively]. These findings support the premise of this case report which describes an individual psychotherapy intervention aimed at reducing defeatist beliefs and increasing work-related self-efficacy.

Engaging individuals living with SMI in vocational interventions can have multiple barriers; however, improving accessibility of resources, tailoring interventions to an individual’s needs, and finding appropriate resources is helpful (Van Laarhoven et al., 2018). Given that technology is becoming increasingly convenient, generally inexpensive, and widespread, efforts have increased to utilize technology in support of vocational training programs. A recent meta-analysis suggested practitioners wishing to incorporate technology support in vocational interventions should consider one of the most widely evaluated approaches, video modeling (Wicker et al., 2022). Video modeling involves teaching a targeted behavior or skill through the use of a video recording, usually of an individual other than the learner engaging in the targeted skill or behavior (Franzone and Collet-Klingenberg, 2008). Video modeling been integrated across many different technological devices such as, tablets, cell phones, and computers, thus increasing reach and accessibility of career interventions. Use of video modeling has shown promise in assisting with the acquisition of vocational skills, such as clerical skills, communication, and professionalism (Alexander et al., 2013; Cullen et al., 2017; Heider et al., 2019; Connor et al., 2020). Use of this technology may also help increase job-search self-efficacy, motivation, and job-search activities (Bahcali and Ozen, 2019), all important areas to consider when providing vocational rehabilitation to individuals living with SMI.

## Material and methods

This retrospective case report took place at a Veteran Affairs (VA) medical center, with multiple services provided including therapy, case management, psychiatric services, and supported employment. This case report focuses on weekly 50-minute therapy session across a 4 month-period in which the client was able to utilize video modeling both in session and at home to take steps toward employment goals.

### Procedure

The primary procedure explored in this case report began after initial cognitive approaches (Beck, 2020) (e.g., thought records) yielded limited results, related to the client’s belief that they weren’t capable of completing basic job-tasks. As a result, the therapist saw an opportunity to use modeling techniques to enhance the client’s perception of their vocational capability. This case report examines the promotion of self-efficacy through use of YouTube, an online video sharing platform. The clinician would navigate to YouTube.com during sessions to demonstrate the steps to search for a pre-existing video that offered step-by-step instructions on how to accomplish a specific work-related task (e.g., How to use a case step-by-step” or “How to stock shelves”). The client would then repeat the steps and navigate on his own. Further, during session when the client would be stuck during a cognitive restructuring exercise related to vocational goals or would express a self-defeating belief (e.g., cognitive distortion) regarding his ability to complete a specific behavior or skill related to work, the clinician would cue the client to explore this belief further. The exploration involved indicating his distress level when imagining himself engaging in the task. Then the client would navigate to YouTube, devise a search term to explore his belief (e.g., how to bag groceries), view a short video, then indicate his new SUDS score and comment about this experience, then express a balanced thought to replace the self-defeatist thought. Other procedures during treatment comprised of:

- Identifying challenging symptoms (e.g., auditory hallucinations, self-defeating beliefs)
- Improving hygiene through charting (e.g., shower, brush teeth twice daily)
- Cognitively restructuring responses to job opportunities
- Developing computer skills (e.g., establish email and familiarize with internet searching)
- Selecting and using coping skills (e.g., breathing to cope, walking)
- Utilizing supported employment to create a resume and search for job opportunities
- Preparing for and perform interview role plays
- Creating a plan to prevent and manage challenging feelings and disruptive behaviors

### Measures

To capture formative evaluation information, the client completed two self-report scales throughout treatment. The first, the Beck Depression Inventory (BDI-II), consists of 21-items assessing somatic, cognitive, and affective issues that lead to an overall rating of the severity of depressive symptoms (possible score range 0–63); higher scores indicating more depressive symptoms (Beck et al., 1996). The BDI-II was administered each month of treatment. The Subjective Units of Distress Scale (SUDS) (Wolpe and Lazarus, 1966; McCabe, 2015) was verbally administered before and after video modeling and is a widely utilized rating scale that assesses an individual’s level of distress and intensity of feelings; the scale ranged from 0 (total relief) to 10 (unbearably overwhelmed by emotions).

## Theory/calculation

Cognitive therapy served as the overarching initial psychotherapy framework. Cognitive therapy emphasizes how belief distortions (e.g., overgeneralization and selective abstraction) are related to and maintain psychopathology (Beck, 2005)). From this theoretical lens, the psychotherapeutic process focused on cognitive techniques (e.g., cognitive restructuring) for reducing or managing the client’s dysfunctional beliefs. As progress was stalled, Bandura’s theory of self-efficacy (Bandura, 1977), was integrated into this psychotherapeutic to not only reduce defeatists beliefs, but also enhance work-related self--efficacy. Self-efficacy refers to a person’s belief about their ability to perform a task associated with a specific situation and is derived from four sources: performance accomplishments (e.g., real-life experience completing a task), vicarious experience (e.g., observing others completing a task), verbal persuasion (e.g., other people suggesting one can succeed at a task), and emotional arousal (e.g., affective states relay information about one’s abilities). Further, for this case, increased self-efficacy appeared contingent on the acceptance and adoption of technology, specifically YouTube.

The unified theory of acceptance and use of technology (UTAUT) incorporates multiple theories including social cognitive theory (SCT) to predict technology adoption (Venkatesh et al., 2003). SCT provides assumptions about how to guide and motivate individuals toward personal change (Bandura, 1986), and emphasizes the role of perceived self-efficacy as a key factor that helps to explain human agency (Bandura, 2001a). The four constructs within UTAUT are performance expectancy, effort expectancy, social influence and facilitating conditions; together incorporating determinants of intent and the behavior of the user (Venkatesh et al., 2003). Moreover, these predictors are moderated by age, gender, experience and voluntariness of use. In practice, utilizing this model to increase the likelihood of usage and adoption may include guidance from the therapist across UTAUT constructs. For example, 1) informing the client about how this technology can be helpful (performance expectancy), 2) demonstrating use to show the degree of ease required to utilize the technology (effort expectancy), 3) explaining how widely used the technology is and the influence and importance it holds to others (social influence) and, 4) explaining how it can be accessed across settings and modalities including on the their phone, home computer, and outside of session (facilitating conditions). Integrating technology in treatment, through the use of step-by-step YouTube videos, represents a practical and accessible application of UTAUT and self-efficacy theory to enhance self-efficacy expectations by providing the client with vicarious experiences (e.g., observing others perform work-related tasks).

## Results

### Case introduction

This case explores the treatment of Carl (name changed), a 55-year-old White male Veteran with a primary diagnosis of schizoaffective disorder who was treated in a VA facility over several years. Carl was divorced and has meaningful relationships with his adult children and ex-wife. He lived in different housing settings over the years including subsidized group housing. He had goals of living independently and attaining employment.

### Presenting complaints

Upon initial presentation and individual psychotherapy, Carl experienced auditory hallucinations, paranoia, loud, disorganized, tangential, racing thoughts, pressured speech, and feelings of euphoria. After the first two-years in care, his predominate presentation consisted of feelings of sadness, reduced interest in activity, decreased appetite, lack of energy, lack of concentration, low motivation, and lack of personal-hygiene (e.g., changing clothes, brushing teeth). During times in which he experienced low mood for an extended period, he reported auditory hallucinations that consisted of disparaging remarks, which interfered with his ability to navigate daily activities.

### Case conceptualization

Carl struggled with self-defeating beliefs that interfered with his ability to initiate activity toward his goals, specifically taking steps toward employment and independent housing. Rumination about failures in his life (e.g., being terminated from his job and divorce) fueled this belief and these thoughts were activated when he initiated a new activity. Moreover, when Carl did take steps toward his goals, he did not perceive these efforts as effective which diminished his ability to build self-efficacy. Further, his inaction and inability to recognize progress bolstered self-defeatist beliefs and created a cycle of inaction that propagated negative beliefs about himself (e.g., “I’m not capable”).

### Brief history, course treatment and assessment

Carl worked as a skilled manufacturer for over 20 years and was the primary provider for his family. He had two psychiatric hospitalizations four-years apart while he was employed, but first sought care at a VA facility several years after being terminated from his job. Carl first presented for acute psychiatric care. After his inpatient stay and five years after initial presentation to a VA facility, Carl accepted a referral for individual psychotherapy that lasted four months. Carl also received services from a multidisciplinary team that included supported employment, case management, psychiatric services, and medication management (i.e., Lurasidone HCL 20 mg, Apriprazole 20 mg, Buproprion 30 mg, Risperidone 4.5 mg). Treatment methods aligned with psychosocial rehabilitation principles, prioritizing a commitment to recovery based on an objective restoration of functioning, which leveraged the client’s strengths and utilized a multi-disciplinary approach based on the clients stated goals (King et al., 2013). Moreover, a key focus of treatment was emotional changes (i.e., perceived intensity of negative emotions) through vicarious learning processes.

**Fig. 1.**
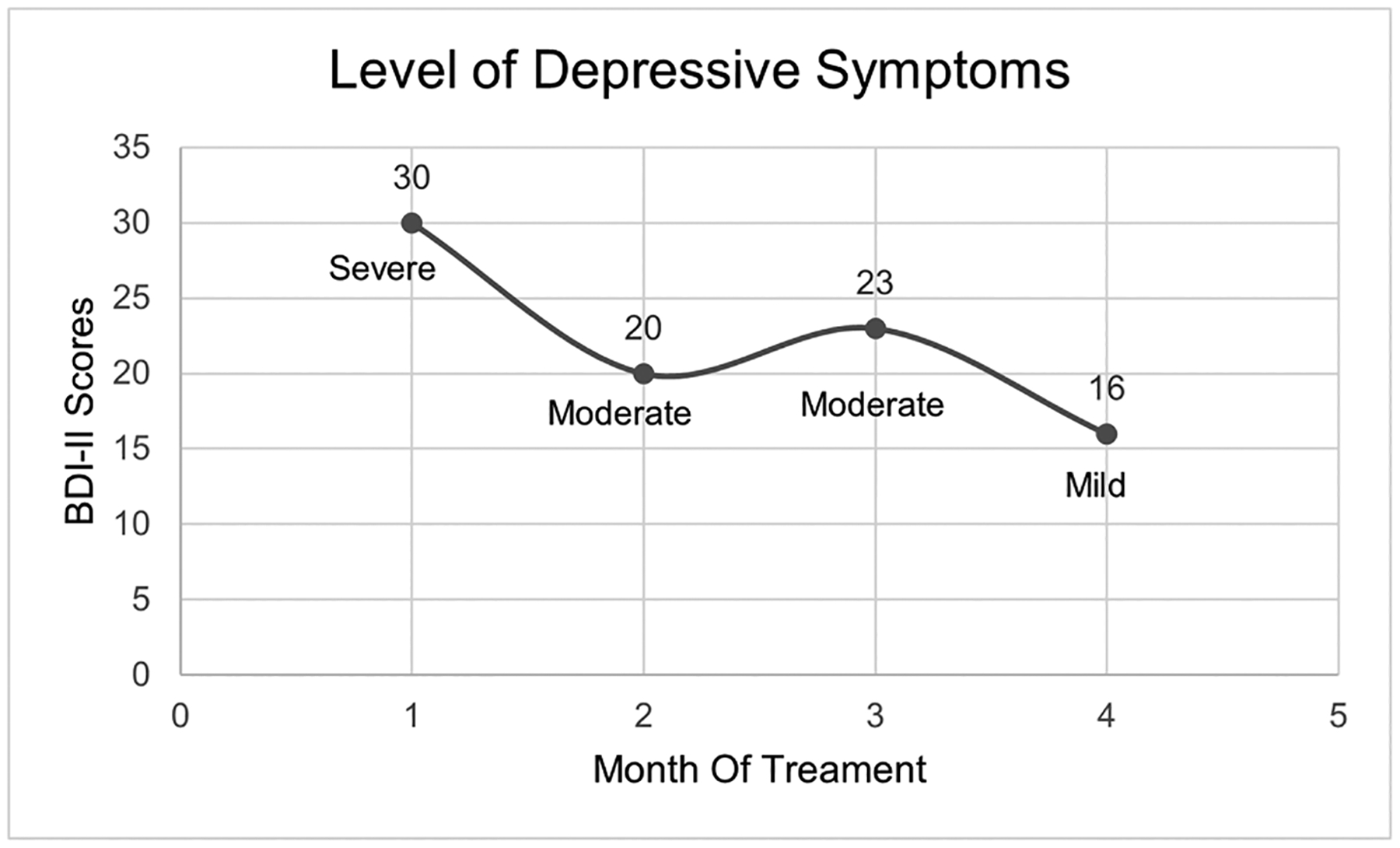
Depressive symptoms throughout treatment using video modeling.

Carl first engaged in individual psychotherapy several months after the death of a family member. Carl’s bereavement process was explored, and depressive symptoms were captured. After the first month of treatment, Carl scored a 30 on the BDI-II (Fig. 1) indicating severe depressive symptoms. Carl explained that he experienced depressive symptoms prior to the death of a close family member, but that challenges around this loss led him to accept a referral for individual psychotherapy. Work began by using a shared decision-making model of care (Drake et al., 2022) first addressing Carl’s immediate felt needs (i.e., increasing more contact with his adult children and personal hygiene), then psychoeducation around schizoaffective disorder and available evidence for specific treatments. Carl and the clinician collaboratively built goals and a treatment plan. Over the course of treatment, session time was split to accomplish several related goals. Part of session was dedicated to identifying symptoms that interfered with goals, learning to engage in cognitive restructuring techniques and physical coping skills (e.g., walking, social activity, breathing exercises, to manage symptoms and improve functioning. Eventually, this culminated in the creation of a Wellness Recovery Action Plan^™^ (WRAP), an individualized plan to achieve wellness (Copeland, 2001). Other parts of session were focused on steps to identify, apply, interview, and be hired for a job.

When exploring the application and interview process for attaining employment he explained “I can’t do that…I won’t be able to do the job… I’ll be let go again…” These beliefs interfered with both taking even basic steps toward searching for employment and with initial cognitive restructuring practices. Whether it was stocking shelves or performing complex manufacturing tasks, the thoughts of engaging in these activities were accompanied by negative beliefs, feeling overwhelmed, and inaction. To Carl, unfamiliar behaviors or skills related to work were seen as insurmountable. During session the clinician and Carl discussed ways to both increase awareness about employment opportunities and learn about the skillset required to accomplish specific jobs. This led to in-session discussion about 1) how to navigate to the internet, 2) examine job opportunities online, and 3) use YouTube video modeling to view step-by-step videos that show the user how to complete specific tasks.

During the teaching process the clinician would navigate to YouTube to demonstrate the steps to search for a video, then have Carl navigate on his own. This process was repeated with job search sites as well. Further, during session when Carl indicated a belief that he was unable to accomplish a task, the clinician would cue Carl to explore this belief further. Carl would then indicate the distress when imagining himself engaging in the job task through verbally indicating his current SUDS score. Then Carl would navigate to YouTube, devise a search term to explore his belief, view a video, then indicate his new SUDS score.

**Fig. 2.**
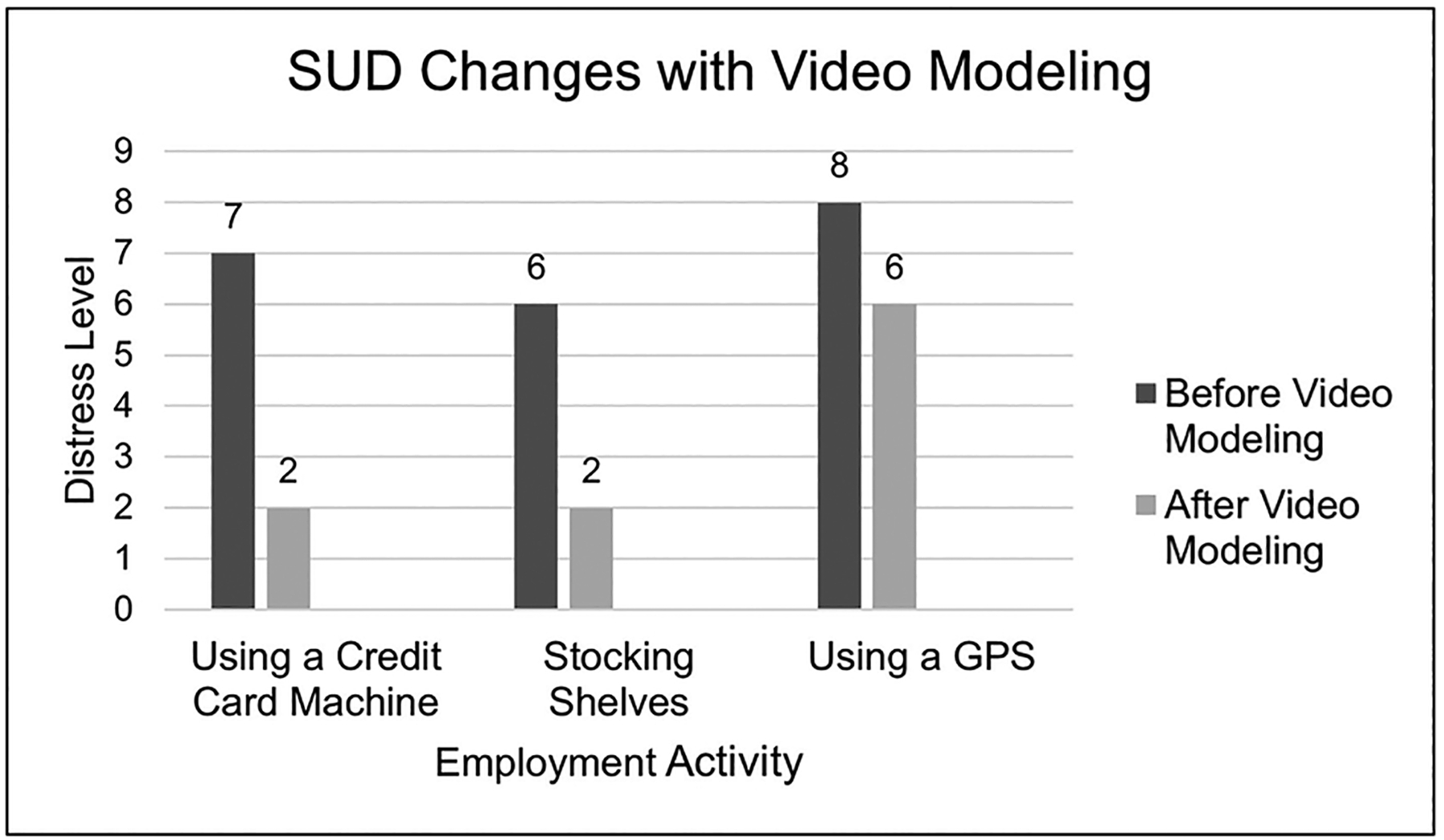
Changes in distress level before and after video modeling.

For example, when Carl discussed the possibility of working a cash register, he explained that he could “never work a credit card machine to ring someone-up…I couldn’t do it, it’s complicated, all these younger people know more…how can I compete,” thus dismissing all jobs that required this function. The clinician cued Carl to navigate to YouTube. Carl then devised his own search phrase “how to use a credit card machine,” selected a short video (under 5-minutes), and upon watching the video Carl appeared to re-examine his belief and explained “I could do that after a couple tries.” His-SUDS score dropped 5 units (Fig. 2). Carl would use this strategy multiple times per session and after several sessions did not require a cue to challenge his belief. He was assigned different tasks related to using YouTube outside of session. After several sessions he reported using YouTube for other activities and reported searching “how to use a camera to take a good photo” and subsequently joined a photography group.

Over time, depressive symptoms decreased from severe (BDI-II score of 30) in Month One, to mild symptoms (BDI-II score of 16) in Month Four (Fig. 1). In Month Four, Carl watched a YouTube video about “how to apply to a job” and explained “Even if it doesn’t work out, I’ll be fine and just apply to more…” Carl increased his involvement in supported employment services, created a resumé, and searched for jobs online. He applied to and interviewed for a part-time job in the manufacturing business. The clinician and Carl ended work together subsequent to the clinicians new role, but Carl continued his job search, involvement with supported employment and continued to reference his WRAP^™^ as needed. One month after the conclusion of therapy he began volunteering. Several months later, he began working part-time as a gift wrapper, and within a year was able to move out of his group home and live independently.

## Discussion

Results of this case report show a promising preliminary understanding of how readily available internet resources can contribute to increased work readiness and openness to work experiences. This case also serves as an example of how vicarious learning activities may enhance work readiness for individuals living with SMI and could increase willingness to engage in well-established interventions (e.g., IPS-SE). Interventions by the clinician included utilizing cognitive restructuring, teaching basic computer skills in the process of facilitating employment-related activities (e.g., using email, searching on the internet for job opportunities), and video modeling. All interventions may have contributed to the client’s self-efficacy and were utilized outside of session. Further, this also shows an example of how video modeling facilitated cognitive techniques (i.e. cognitive restructuring) and unlocked the accessibility of a more balanced thought and belief in his abilities.

Use of technology to overcome avoidance-related anxiety to assist with goal attainment is a growing and important field for clinical care. Use of videos for vicarious learning has been found to be effective in changing beliefs and knowledge about feeding practices (Ledoux et al., 2018), speech therapy adherence (van Leer and Connor, 2015), and adopting health behaviors among chronic pain patients (Schweier et al., 2014). Other types of technologies such as virtual reality and augmented reality have also been found to positively impact not only skills acquisition but also cognitive and affective domains such as self-efficacy and motivation (Ding et al., 2020; Freitas et al., 2021). These technologies can allow clients to be scaffolded into revising their perceived threat and their perceived efficacy.

Therapists are often tasked with utilizing multiple tools for a gradated therapeutic experience. In the case of Carl, high perceived threat, high distress, and low perceived self-efficacy resulted in an automatic response to catastrophize and avoid attempting solutions. By using a low cost, highly accessible online resource such as YouTube, both the therapist and Carl had access to a widespread and free technology strategy to help Carl overcome his initial avoidance and high distress. Vicarious learning through videos supported Carl in cultivating workforce skills and self-efficacy prior to opportunities for real-world practice.

## Conclusion and future direction

New tasks and challenges can be difficult for anyone attempting to join the workforce. Basic computer skills training as well as task education through easily accessible video modeling may facilitate confidence to approach challenges. This intervention may also have future benefits. The client now has basic computer skills to utilize in social, intellectual, and other domains, as well as knowledge on accessing YouTube to manage stressful emotions related to prospective challenges. Integrating technology to target defeatist beliefs may bolster work readiness, however this should be further explored. In general, utilizing YouTube or similar low-cost, high access resources could be implemented in a variety of settings to bolster recovery efforts for persons with SMI.

## Data Availability

Due to protection of anonymity the materials are not available.
